# Housing inadequacy in rural Saskatchewan First Nation communities

**DOI:** 10.1371/journal.pgph.0000470

**Published:** 2022-08-02

**Authors:** Shelley Kirychuk, Eric Russell, Donna Rennie, Chandima Karunanayake, Clarice Roberts, Jeremy Seeseequasis, Brooke Thompson, Kathleen McMullin, Vivian R. Ramsden, Mark Fenton, Sylvia Abonyi, Punam Pahwa, James A. Dosman

**Affiliations:** 1 Canadian Centre for Health and Safety in Agriculture, University of Saskatchewan, Saskatchewan, Canada; 2 Department of Medicine, University of Saskatchewan, Saskatchewan, Canada; 3 Community Member, Montreal Lake Cree Nation, Saskatchewan, Canada; 4 Community Member, Beardy’s and Okemasis First Nation, Saskatchewan, Canada; 5 Community Health and Epidemiology, University of Saskatchewan, Saskatchewan, Canada; 6 Department of Academic Family Medicine, University of Saskatchewan, Saskatchewan, Canada; 7 Division of Respirology, Critical Care and Sleep Medicine, University of Saskatchewan, Saskatchewan, Canada; Queen’s University, CANADA

## Abstract

Housing and house conditions on First Nation communities in Canada are important determinants of health for community members. Little is known about rural First Nation housing in the Canadian Prairies. The aim was to survey houses in two rural First Nation communities in Saskatchewan, Canada to understand housing conditions, prevalence of mold/mildew and dampness, and sources, locations and frequency of mold and dampness. Surveys were conducted with an adult member of each household in 144 houses. Surveys assessed: size, age, and number of rooms in the house; number of individuals residing in the house; presence of mold/mildew and dampness, and sources, locations and frequency of mold and dampness. Houses were mostly two-bedrooms (25.7%) or more (67.4%). Thirty-one percent of houses had six or more people living in the house with crowding present in 68.8% of houses. Almost half of the houses (44.5%) were in need of major repairs. More than half of the houses had water or dampness in the past 12 months in which dripping/puddles and standing water were most commonly identified and were from surface water and plumbing. More than half of the houses indicated that this dampness caused damage. A smell of mold or mildew was present in over half of the houses (52.1%) and 73.3% of these houses indicated that this smell was always present. Housing adequacy including crowding, dampness, and mold are significant issues for houses in these two rural Saskatchewan First Nation communities. Housing inadequacy is more common in these rural communities as compared to Canadian statistics. Housing inadequacy is modifiable and is important to address for multiple reasons, but notably, as a social determinant of health. Federal government strategy to address and redress housing in First Nation communities in Canada is a fiduciary responsibility and critical to reconciliation.

## Introduction

Housing is a social determinant of health [1–7] and housing is a significant issue for many First Nation communities [8–12] in Canada. Many Canadian First Nation families live in a house that is not suitable including inadequate house size for the occupant composition, house in need of major repairs, and dampness and mold issues [8–23]. Respiratory illness disproportionately affects First Nation populations in Canada in which some of the associated factors include economic, environmental, social and historic factors; with housing being an important environmental factor associated with respiratory illness [17–31]. There is almost no data or research on housing conditions specific to rural First Nations communities in Canada, and none in Saskatchewan. The purpose of this paper is to highlight the nature of house characteristics and conditions in two rural First Nation communities in Saskatchewan, Canada.

## Methods

### Ethics statement

The research methods were approved by the University of Saskatchewan Biomedical Research Ethics Board (Bio#12–189). Written informed consent was obtained from the household member completing the questionnaire.

### Methods conceptualization, surveys

Community members were involved in the conceptualization, development, collection, and interpretation of the surveys and data [32, 33]. Housing surveys were undertaken with the two communities to understand the housing factors in the community as a component of the larger study evaluating the respiratory health of community members [32]. The two First Nation communities are located in north central Saskatchewan with populations between 2000–3000 people and are at least a 90 km distance from the nearest city. House surveys were collected from 2013 to 2014 as part of Phase 1 of a longitudinal study of respiratory health of residents in two First Nation communities in Saskatchewan in which the details and study methodology have been indicated elsewhere [32, 33]. All households in the two communities were invited to participate and 406 households completed the Phase 1 respiratory health questionnaires and assessments [32]. Those 406 households could indicate, as part of the Phase 1 questionnaire assessment, if they wished to have further house assessments. Those households indicating ‘yes’ were invited to participate in this house study.

The house evaulation consisted of a survey which included the type of house, age, number of rooms, number of bedrooms, number of people currently living in the house, type of heating, air exchange options, dampness in the house, if there were signs and/or smell of mold/mildew and if the house was in need of minor and/or major repairs. If dampness or mold were indicated, further questions assessed location, source, level and duration.

## Results

### House characteristics

Surveys completed were from a total of 144 houses in the two rural Saskatchewan First Nations communities with surveys from 59/321 houses in Community A and 85/259 houses from Community B. All houses surveyed were single-detached family dwellings. House characteristics describe the house in terms of age, number of rooms, number of people and amenities including humidifiers, air conditioners, dryer, heating, ventilation, etc., are shown in Table 1. The majority of houses were 3-bedroom units. The mean size of houses was 964 sq.ft (SD 211 sq.ft; range:400–2400 sq.ft). Only one house was larger than 1440 sq.ft. Twenty-six percent of houses were 2-bedroom while the majority of houses were 3–5 bedroom houses (67.4%). The age of the house differed by community with more houses in Community B being built after 1990. Thirty-one percent of houses had six or more persons residing in the house and close to two-thirds of the houses had four or more residents.

**Table 1.** Housing characteristics of two Saskatchewan First Nation communities.

	n (%)
Type of Dwelling	
2 bedroom house	37 (25.7)
3–5 bedroom house	97 (67.4)
Bi-level	3 (2.1)
Trailer	4 (2.7)
1-bedroom	3 (2.1)
Basement/Crawl space	
Below grade crawl space	50 (34.7)
Basement	65 (45.1)
None/above grade crawl space	27 (18.7)
Don’t know/missing	2 (1.5)
Age of house	
1990 or later	85 (59.0)
1969–1989	47 (32.6)
1949–1969	11 (7.6)
Don’t know/missing	1 (0.8)
Number of people in house	
1	8 (5.5)
2	7 (4.9)
3	35 (24.3)
4	31 (21.5)
5	17 (11.8)
Six or more	44 (30.6)
Don’t know/missing	2 (1.4)
Humidifier	
Yes	13 (9.0)
No/Don’t know	131 (91.0)
Air Conditioner	
Yes	31 (21.5)
No/Don/t know	113 (78.5)
Unvented dryer	
Yes	11 (7.6)
No	133 (92.4)
Sump pump	
Yes	73 (50.7)
No	71 (49.3)
Main source of heating	
Gas	131 (91.0)
Oil	9 (6.2)
Electric	4 (2.8)
Filter on heating system	
Yes	132 (91.7)
No/ Don’t know	12 (8.3)
Heat Recovery ventilation	
Yes	71 (50.0)
No	66 (46.5)
Don’t know	5 (3.5)

### House conditions

**Fig 1:**
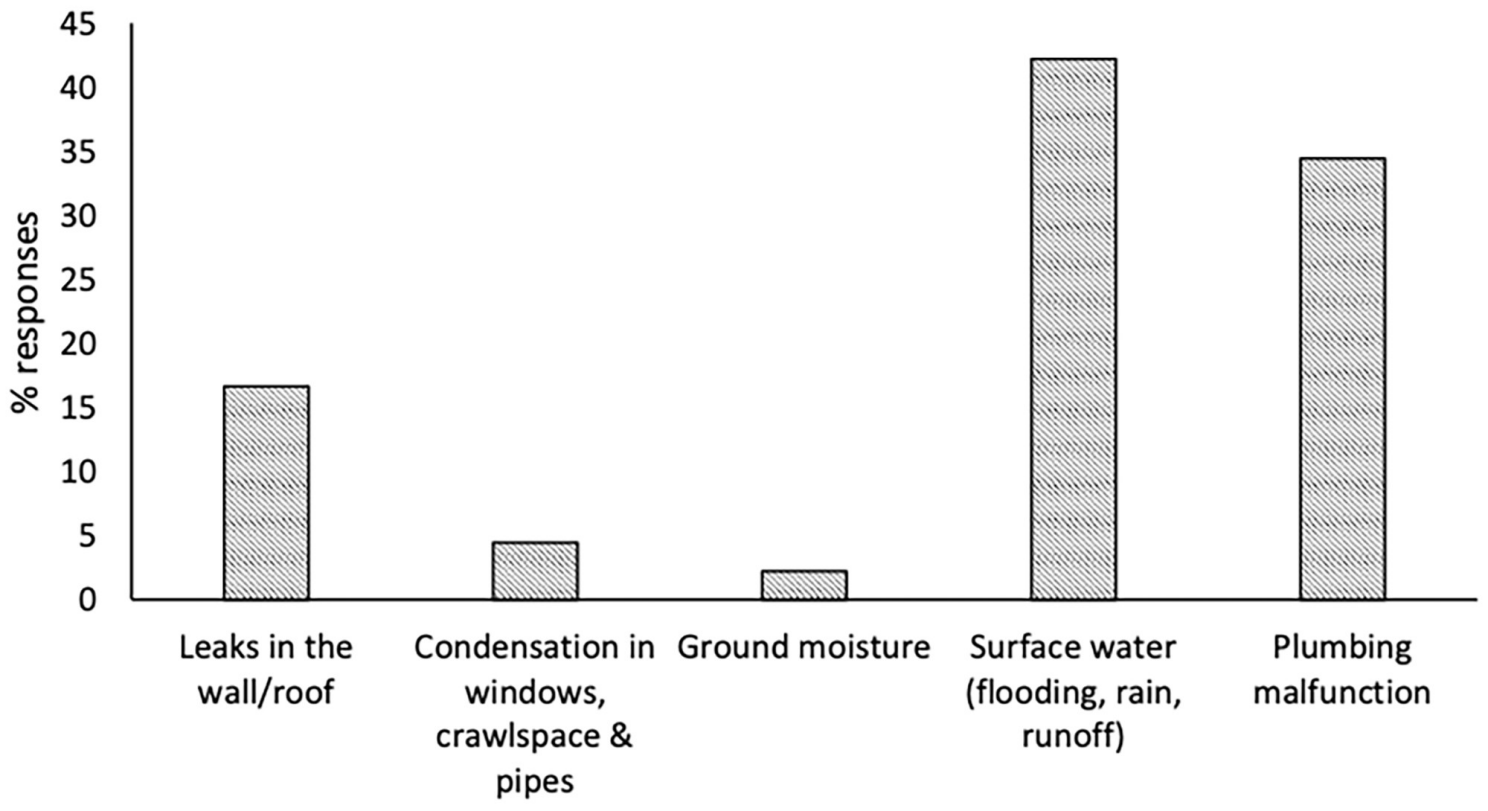
Primary sources of water damage in house (n = 90).

**Fig 2:**
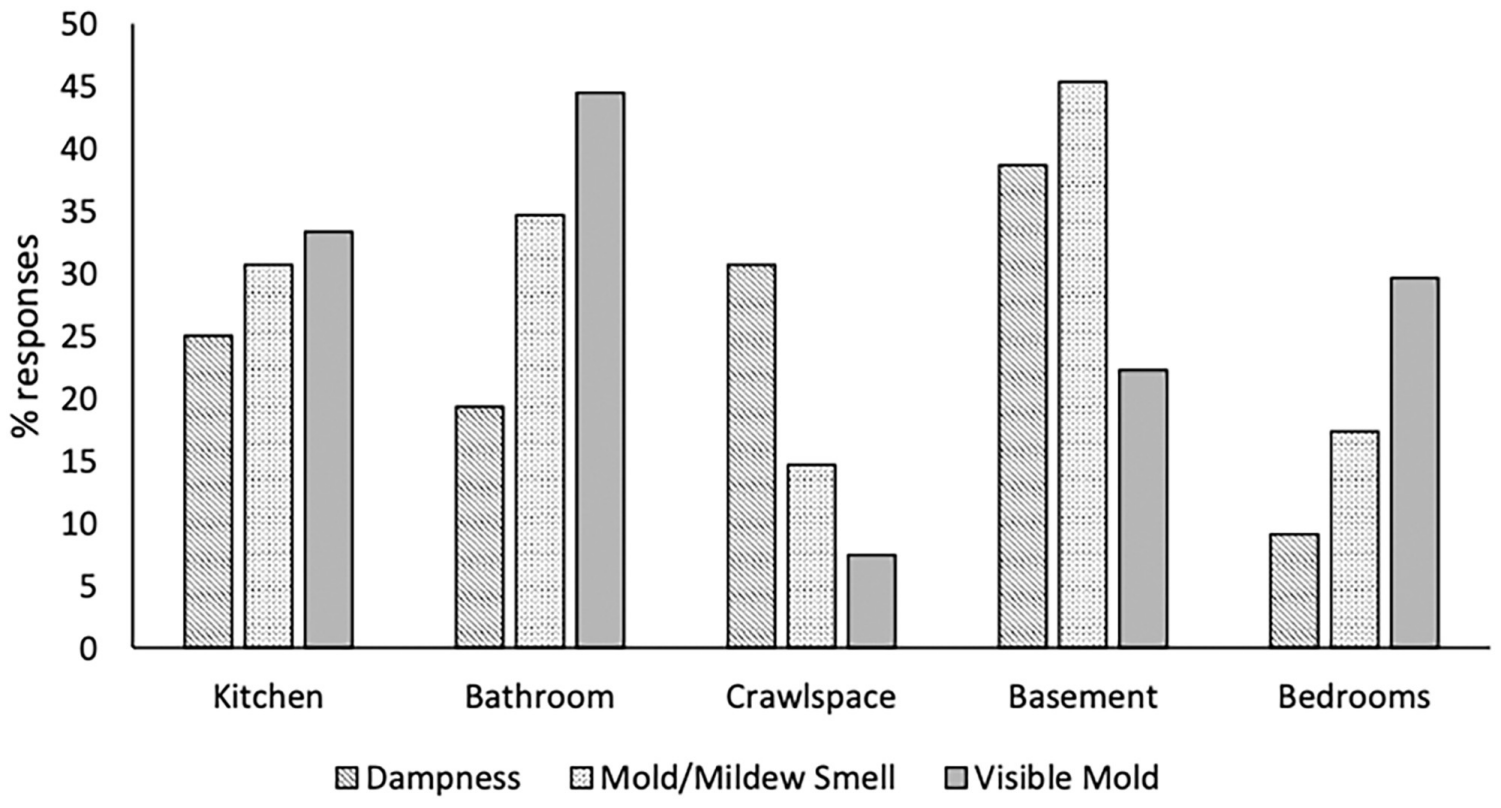
Location of conditions (dampness, smell of mold, visible mold) in the house.

House conditions describe the condition of the house in terms of crowding, repairs required, and dampness and mold, and are shown in Table 2. Crowding is present in almost 70% of the houses, meaning that in the majority of houses there was more than 1 person per bedroom, two or more people per bedroom was present in 21.6% of the houses, while 5.6% of these houses had more than 2 people per bedroom. Almost half of the houses (44.8%) were in need of major repairs. Over half of the houses (62.2%) indicated water or dampness in the house within the past year while standing water and dripping/puddles were the most commonly indicated type of water/dampness in the house, and 13.5% indicated that this dampness was present almost all the time. Almost half of the houses (47.2%) indicated that there had been damage caused by this dampness. The primary source of water damage in the house was from surface water and plumbing (Fig 1). Over half of the houses (52.1%) indicated having a smell of mold or mildew present in the house and 73.3% of these houses indicated that this smell was always present. In over half of the houses (56.2%), visible mold in the house was indicated. Dampness, visible mold, and mold/mildew smell were found throughout the house (Fig 2).

**Table 2.** Housing conditions in two Saskatchewan First Nation communities.

	n (%)
Crowding	
< = 1 person/bedroom	44 (30.6)
> 1 person/bedroom	99 (68.8)
Missing	1 (0.6)
House in need of repairs	
Minor	56 (39.2)
Major	64 (44.8)
Maintenance only	23 (16.1)
Water or dampness in home in past 12 months	
Yes	89 (62.2)
No	54 (37.8)
*Type of water/dampness in the past 12 months*	
Damp	7 (4.9)
Dripping/Puddles	44 (30.6)
Standing water	38 (26.4)
None	55 (38.2)
*Frequency of dampness*	
Almost all the time	12 (13.5)
Frequently (e.g. on most rainy days)	10 (11.2)
Occasionally (e.g. only during heavy rains)	39 (43.8)
Rarely	25 (28.1)
Don’t know	3 (3.4)
Damage caused by dampness	
Yes	68 (47.2)
No	66 (45.8)
Don’t know/missing	10 (7.4)
Smell of mold or mildew	
Yes	75 (52.1)
No/Don’t know/Missing	64 (44.4)
Don’t know/missing	5 (3.5)
*Frequency of mold or mildew smell*	
Always	55 (73.3)
Less than 6 months/year	18 (24.0)
Don’t know	2 (2.7)
Visible mold or mildew	
Yes	81 (56.2)
No	56 (38.9)
Don’t know/missing	7 (4.9)

## Discussion

These two rural Saskatchewan First Nation communities more frequently had housing conditions that were not suitable, and the conditions were not as good as that indicated at the National level for both Indigenous and non-Indigenous people in Canada. The results revealed that houses on these two rural Saskatchewan First Nation communities are small single detached houses in which large numbers of individuals were dwelling. The majority of houses surveyed indicated the house was in need of major repairs. Dampness, damage caused by dampness, visible mold, and a mold or mildew smell present in the house were common. Of the houses in which a moldy smell was present almost three quarters further indicated that the smell was almost always present.

All of the houses surveyed in these two rural Saskatchewan First Nations were single detached houses with the majority of houses having 3–5 bedrooms. This is higher than the 2016 Canada census in which 53.6% of dwellings in Canada are single-detached houses, and in Saskatchewan it is 72.6% [11]. In general in rural Canada, and particularly in rural Saskatchewan, one would find predominanatly single-detached houses.

Results revealed that 31% of houses surveyed had six or more persons residing in the house and close to two-thirds of houses had four or more residents. Canada Mortgage and Housing Corporation’s (CMHC) National Occupancy Standard is a determinant of housing suitability, or crowding, a measure of whether the house had enough bedrooms for the composition and number of residents [12]. According to the 2016 census, 18.3% of the Canadian Indigenous population lived in housing that was not suitable for the number of people who lived there [8, 11–14]. These two rural First Nation communities had a greater percentage of residents living in crowded houses as compared to the national average. Crowding in houses has been associated with increased respiratory risk [22, 23], and an important determinant of housing adequacy [12, 13]. The number of people that reside in the house and the number of people per bedroom reflect a First Nation kinship model in which household units go beyond the traditional European nuclear family unit. The housing standards and Federal government supports for First Nation housing are based on a colonial model, do not reflect First Nations reality, and this significantly contributes to housing inadequacy. Further, housing is a finite resource in these two communities. The complexities of attaining new Band houses and member-owned houses significantly impairs new builds and suitable builds. Addressing housing suitability in rural First Nations communities is important to improving health outcomes with residents in the houses.

In addition to crowding, the majority of the houses surveyed would be considered to have housing inadequacy due to a house in need of major repairs, mold and smell of mold in the house, water or dampness in the house, and these house conditions have been associated with health outcomes of occupants [24–33]. Almost half of the houses surveyed in this study indicated the house was in need of major repairs. The 2016 Canada census revealed that for Aboriginal people in Canada about two in ten houses required major renovations [11]. From the 2006 Aboriginal Peoples Survey, living in a house in need of major repairs was found to be associated with an increase of ever asthma in both Aboriginal adults and children living off reserves [23]. This is higher than that reported in a study of school age non-Aboriginal children living in rural Saskatchewan that showed 27.9% of rural children were living in houses with dampness and 19.5% indicated living in houses with mold or mildew [31]. Dampness and mold were common in the houses surveyed with over half of the houses having mold and dampness, which is higher than mold levels reported in non-Aborignal rural homes in Saskatchewan [28, 31]. Mold and dampness have been associated with health outcomes and linked to upper and lower respiratory conditions in adults and children in both Indigenous and non-Indigenous populations [23–31]. Respiratory illness disproportionately effects First Nation peoples in Canada with house conditions known to be an important environmental determinant of health [1–12]. Housing inadequacy in these two First Nation communities is well above the National and Provincial rates. Economic, environmental (including housing), social, and historic factors, which differ regionally and between rural and urban settings, has been shown to be important to health outcomes of First Nation people. Federal support for housing and housing repairs at the First Nation level leaves First Nations Bands unable to address these issues. Addressing housing inadequacy in rural First Nation communities is important.

The strengths of this work are: the community driven research approach and data collection; a focus on two rural First Nation communities, and; a population-based survey approach. A limitation to this work is that the house evaluations occurred in 30% of the households and therefore, it is not possible to generalize our results to the community level.

Housing inadequacy, including crowding, homes in need of major repairs, mold and smell of mold and the associated respiratory health effects in the residents in the houses are a reflection of a colonial housing model that does not fit with the realities of these two First Nations. Housing, as a social determinant of health, can be defined as a condition that results in health inequalities that are unfair or unjust and do not meet fiduciary obligations or international human rights [34, 35]. Canadian policy statements speak to the disparities in First Nation housing and the need to address these disparities to reduce health inequalities [1, 36–41], however, real National level impact that redresses First Nation housing in Canada is slow to be realized. Local initiatives and partnerships are developing, including the work undertaken in one of these communities [42], however, a dedicated Federal government response is required. In Canada, housing, a modifiable condition, should not be a contributor to illness. The Federal government has the inherent responsibility to address and redress First Nation housing, including recognizing the role of colonization and Federal government housing support practices for First Nations, as important contributors to the current state. Strategies such as the Assembly of First Nations’ (AFN) “Rights-based Approach” framework [43] that visions, transitions, and supports First Nation control and management of housing, and in which Treaty, Indigenous and human rights to safe houses is met and maintained by First Nations is an imperative step to health, reconciliation, and decolonization.
